# Variability and Predictors of Urinary Bisphenol A Concentrations during Pregnancy

**DOI:** 10.1289/ehp.1002366

**Published:** 2010-10-08

**Authors:** Joe M. Braun, Amy E. Kalkbrenner, Antonia M. Calafat, John T. Bernert, Xiaoyun Ye, Manori J. Silva, Dana Boyd Barr, Sheela Sathyanarayana, Bruce P. Lanphear

**Affiliations:** 1 Department of Environmental Health, Harvard University, Boston, Massachusetts, USA; 2 Department of Epidemiology, University of North Carolina–Chapel Hill, Chapel Hill, North Carolina, USA; 3 National Center for Environmental Health, Centers for Disease Control and Prevention, Atlanta, Georgia, USA; 4 Rollins School of Public Health, Emory University, Atlanta, Georgia, USA; 5 Department of Pediatrics, University of Washington/Seattle Children’s Hospital, Seattle, Washington, USA; 6 Department of Pediatrics, Division of General and Community Pediatrics, Cincinnati Children’s Hospital Medical Center, Cincinnati, Ohio, USA; 7 Simon Fraser University, Vancouver, British Columbia, Canada

**Keywords:** bisphenol A, dietary, occupational, predictors, pregnancy, prenatal, variability

## Abstract

**Background:**

Prenatal bisphenol A (BPA) exposure may be associated with developmental toxicity, but few studies have examined the variability and predictors of urinary BPA concentrations during pregnancy.

**Objective:**

Our goal was to estimate the variability and predictors of serial urinary BPA concentrations taken during pregnancy.

**Methods:**

We measured BPA concentrations during pregnancy and at birth in three spot urine samples from 389 women. We calculated the intraclass correlation coefficient (ICC) to assess BPA variability and estimated associations between log_10_-transformed urinary BPA concentrations and demographic, occupational, dietary, and environmental factors, using mixed models.

**Results:**

Geometric mean (GM) creatinine-standardized concentrations (micrograms per gram) were 1.7 (16 weeks), 2.0 (26 weeks), and 2.0 (birth). Creatinine-standardized BPA concentrations exhibited low reproducibility (ICC = 0.11). By occupation, cashiers had the highest BPA concentrations (GM: 2.8 μg/g). Consuming canned vegetables at least once a day was associated with higher BPA concentrations (GM = 2.3 μg/g) compared with those consuming no canned vegetables (GM = 1.6 μg/g). BPA concentrations did not vary by consumption of fresh fruits and vegetables, canned fruit, or store-bought fresh and frozen fish. Urinary high-molecular-weight phthalate and serum tobacco smoke metabolite concentrations were positively associated with BPA concentrations.

**Conclusions:**

These results suggest numerous sources of BPA exposure during pregnancy. Etiological studies may need to measure urinary BPA concentrations more than once during pregnancy and adjust for phthalates and tobacco smoke exposures.

Bisphenol A (BPA) is an estrogenic monomer used to produce polycarbonate plastics and resins that can be used in medical equipment, children’s toys, water supply pipes, carbonless paper, cigarette filters, and food container linings (Chapin et al. 2008; Jackson and Darnell 1985). More than 6 billion pounds of BPA were manufactured in 2003, making it one of the highest-volume production chemicals in the world (Burridge 2003). The U.S. population, including children and pregnant women, have nearly ubiquitous exposure to BPA (Calafat et al. 2008; Vandenberg et al. 2010), likely because of its pervasiveness in the environment and its ability to leach from food and beverage containers under conditions of normal use.

Growing concern over BPA exposure is reflected in a report from the National Toxicology Program (NTP) and an ongoing risk assessment of BPA by the U.S. Food and Drug Administration (FDA) (Chapin et al. 2008; FDA 2010; vom Saal et al. 2007). The NTP report and others have concluded that prenatal BPA exposure has the potential to alter neurodevelopmental, reproductive, and metabolic end points throughout the life span (Chapin et al. 2008; Palanza et al. 2008; vom Saal et al. 2007). These end points may be sensitive to prenatal BPA exposure, because the developing fetus may be susceptible to environmental toxicants (Mendola et al. 2002). Consequently, understanding predictors and variability of BPA exposure in pregnancy is essential for future studies of the health effects of BPA.

Although pharmacokinetic studies in humans show that BPA has a biological half-life of < 6 hr (Volkel et al. 2002), recent data suggest that BPA has a longer half-life as well as sources of nonoral exposure and may deposit in fat tissue (Fernandez et al. 2007; Stahlhut et al. 2009; Vandenberg et al. 2010). Because of a variety of factors, including the pharmacokinetic properties of BPA and lifestyle factors, serial urinary BPA concentrations show low to modest correlations over 1–6 months (Mahalingaiah et al. 2008; Nepomnaschy et al. 2009; Teitelbaum et al. 2008). Given the rapid elimination of BPA from the body and low degree of within-person correlation, the timing of urine collection may influence the observed BPA concentration and may fail to reflect the dose of BPA that could be related to health end points.

It is estimated that almost 99% of BPA exposure comes from dietary sources in children (Wilson et al. 2007), but comparable studies have not been conducted in pregnant women or adults. Food containers lined with BPA-based resins are a likely source of exposure, and multiple studies have measured BPA in canned foods (Chapin et al. 2008; Lim et al. 2009; Thomson and Grounds 2005). However, no studies have documented a relationship between canned food consumption and urinary BPA concentrations. Sociodemographic factors may influence food choices and act differentially across various populations (Calafat et al. 2008; He et al. 2009; Ye et al. 2008).

Little is known about the variability and determinants of BPA exposure in pregnant women. Thus, we examined the correlation and predictors of urinary BPA concentrations in three serial samples taken over the latter two-thirds of pregnancy from 389 pregnant women in Cincinnati, Ohio. Specifically, we estimated the within- and between-woman variability of urinary BPA concentrations and examined the association between sociodemographic, occupational, dietary, and environmental factors and urinary BPA concentrations.

## Methods

### Study sample

We used data collected from pregnant women participating in the Health Outcomes and Measures of the Environment Study, an ongoing prospective birth cohort in the Cincinnati, Ohio, metropolitan area designed to examine low-level environmental toxicant exposure (Dietrich et al. 2005). Eligibility criteria and participant recruitment have been described previously (Braun et al. 2009). Of the 1,263 eligible women, 468 enrolled in our study (37%), 67 dropped out before delivery, and 3 had stillbirths. The current analyses were further restricted to 389 mothers who delivered singleton children between March 2003 and January 2006. We excluded one woman whose 26-week BPA concentration (1,250 μg/L) was 3 orders of magnitude higher than the median 26-week BPA concentration.

### Urinary BPA concentration

Women provided spot urine samples around 16 and 26 weeks of gestation and within 24 hr of delivery. Urine was collected in polyethylene containers and stored at −20°C until shipped to the U.S. Centers for Disease Control and Prevention (CDC) for analysis. The concentration of total (free plus conjugated) species of urinary BPA was quantified using modified high-performance liquid chromatography-isotope dilution tandem mass spectrometry (HPLC-MS/MS) analytical methods described previously (Ye et al. 2005). Concentrations below the limit of detection (LOD) of 0.4 μg/L were given a value of 
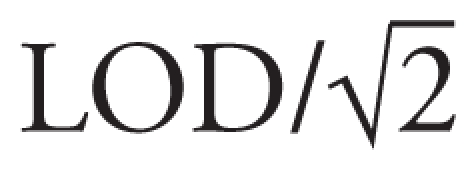
 for the statistical analyses (Hornung and Reed 1990).

Urinary creatinine, measured using previously described methods (Larsen 1972), was used to control for urine dilution. We standardized urinary BPA concentration (micrograms BPA per gram creatinine) to avoid having multiple time-dependent variables in our statistical models.

### Predictors of BPA concentrations

We examined the association between urinary BPA concentrations and demographic, perinatal, occupational, dietary, and environmental variables collected from questionnaires and biological samples. We evaluated demographic and perinatal factors that may be associated with neurodevelopmental outcomes that are typically included as covariates in epidemiological studies. We also examined urinary BPA concentrations according to occupation and diet. Women may be exposed to BPA in occupational settings from BPA-containing medical supplies, food, or cash register receipts. Dietary factors were examined because food is considered the major source of BPA exposure (von Goetz et al. 2010; Wilson et al. 2007). Finally, we examined the association between tobacco smoke and phthalate exposures and urinary BPA concentrations because they may share common sources of exposure [He et al. 2009; National Research Council (NRC) 2008].

Demographic factors included maternal age, education, race, marital status, household income, and occupation. Women self-reported occupation twice for the periods of conception to 20 weeks of gestation and 20 weeks to birth. We categorized women’s occupations using their employer, description of their type of work, and job title. We hierarchically classified women as cashiers, health care workers (e.g., nurse, physical therapist), food service workers (e.g., waitress, cook, fast-food worker), industrial or janitorial workers (factory work or janitor), teachers (including other faculty or staff working in a school), office workers, sales or service workers, other, and unemployed (reference) to avoid having women in more than one occupation category. For example, a woman who worked as a cashier in a fast-food restaurant would be classified as a cashier.

Perinatal and maternal factors included parity, depressive symptoms at 20 weeks of gestation, maternal IQ, and child sex. Parity was abstracted from medical records. Depressive symptoms were measured with the Beck Depression Inventory (BDI-II) (Beck et al. 1996). Maternal IQ was measured using the Wechsler Abbreviated Scales of Intelligence when mothers and children returned for a 1-year postpartum study visit (Wechsler 1999).

Women were interviewed by trained research staff twice during pregnancy about how frequently they consumed certain foods during the periods of conception to 20 weeks and 20 weeks to birth. These foods included store-bought fresh or frozen fish, fresh fruits or vegetables, canned fruits, and canned vegetables. Women also reported the approximate proportion of organic food they ate during the two periods of pregnancy and whether they were strict, partial, or nonvegetarians. These questionnaires were originally designed to assess gestational exposure to mercury and pesticides.

We also examined whether the time of day or fasting status at the time of sample collection influenced urinary BPA concentrations. Fasting status was derived from the time since a woman last consumed any food. We did not include samples taken around birth in these analyses, because the time of day (55%) and fasting time (70%) were missing from a substantial proportion of women.

Serum cotinine and urinary phthalate concentrations were measured in samples collected at 16 and 26 weeks of gestation and within 24 hr of birth. Serum samples were analyzed for cotinine, a biomarker of nicotine exposure, using HPLC-MS/MS (LOD = 0.015 ng/mL) (Bernert et al. 2000). Serum cotinine concentrations were examined as categorical variables [active (> 3 ng/mL), secondhand (0.015–3 ng/mL), or no (< 0.015 ng/mL) exposure] (Benowitz et al. 2009).

We measured nine phthalate metabolites from the same urine samples used to quantify BPA [see Supplemental Material, Table 1 (doi:10.1289/ehp.1002366) for a list of individual phthalate metabolites]. The HPLC-MS/MS analytical methods and quality control procedures used have been described previously (Silva et al. 2007). To simplify our analysis of the phthalates–BPA association, we grouped phthalate metabolites into categories based on the molecular weight of their parent compounds or parent metabolite as follows: low molecular weight (< 250 Da), high molecular weight (> 250 Da), and di(2-ethylhexyl) phthalate (DEHP) metabolites (Engel et al. 2010).

### Statistical analysis

We compared the demographic characteristics of the 389 women with at least one urine measurement with those with all three urine measurements (*n* = 332). Among the 57 women missing one or more urine measurements, 54 had a valid measurement at 16 weeks.

We used two methods to evaluate the reproducibility of urinary BPA concentrations across pregnancy. First, we calculated Pearson correlation coefficients between pairs of BPA and creatinine-standardized BPA concentrations from the 16-week and 26-week and birth urine samples. To determine whether these correlations decayed between pairs of study visits further apart in time, we stratified the Pearson correlation coefficients between log_10_-transformed urinary BPA concentrations taken at 16 and 26 weeks and 26 weeks and birth by the time (in weeks) between measurements.

Next, we calculated intraclass correlation coefficients (ICC) using a one-way random-effects models with unstructured symmetry covariance matrices (Proc Mixed version 9.2; SAS Institute Inc., Cary, NC, USA) to estimate the between- and within-subject variability of urinary BPA, creatinine-standardized BPA, and creatinine concentrations. The ICC can be interpreted as a measure of the reproducibility of the same measurement within an individual. Values can range from 0 (no reproducibility) to 1 (perfect reproducibility) (Rosner 2000). We also calculated these variability measures using just the first two (16- and 26-week) and last two (26-week and birth) urine measurements.

We examined the association between the prenatal urinary BPA concentrations and demographic, perinatal, occupational, dietary, temporal, and environmental factors using linear mixed models because our data involved multiple measurements on the same individual (Fitzmaurice et al. 2004). Demographic and perinatal factors were included as fixed effects in our mixed models. Occupation, dietary, time of sample collection, fasting status, and concentrations of serum cotinine and urinary phthalates metabolites variables were modeled as time-dependent factors to coincide with the temporally relevant urine measurement. Log_10_-transformed creatinine-standardized urinary BPA concentrations (in micrograms BPA per gram creatinine) were the outcome in mixed models with separate models for each predictor. These models were used to calculate the geometric mean (GM) creatinine-standardized urinary BPA concentration by category of predictor variables to appropriately include all three urine measurements. Beta coefficients from the mixed models were exponentiated to produce the ratio of BPA concentrations between categories of predictor variables. Thus, estimates > 1.0 or < 1.0 indicate that the mean creatinine-standardized BPA concentrations were higher/lower for women in that category compared with the reference category. Occupational, dietary, and environmental predictors were additionally adjusted for maternal age, race, education, household income, and marital status.

We conducted secondary analyses that adjusted for urinary dilution by modeling log_10_-transformed urinary creatinine concentrations as a time-dependent covariate instead of using creatinine-standardized BPA concentrations.

### Ethical considerations

The institutional review boards of Cincinnati Children’s Hospital and Medical Center, participating hospitals and obstetric practices, and the CDC approved this study. All mothers provided written informed consent before enrolling in the study.

## Results

Women with all three urinary BPA measurements were more likely to be married (69% vs. 40%), older (30 vs. 27 years of age), and wealthier (median household income: $61,000 vs. $40,000 per year) than women missing one or more measurements. However, 16-week creatinine-standardized urinary BPA concentrations were almost identical among women with all three measurements [GM = 1.9 μg/g; SE of the geometric mean (GSE) = 1.0)] and at least one (GM = 1.9 μg/g; GSE = 1.1) measurement.

**Figure 1:**
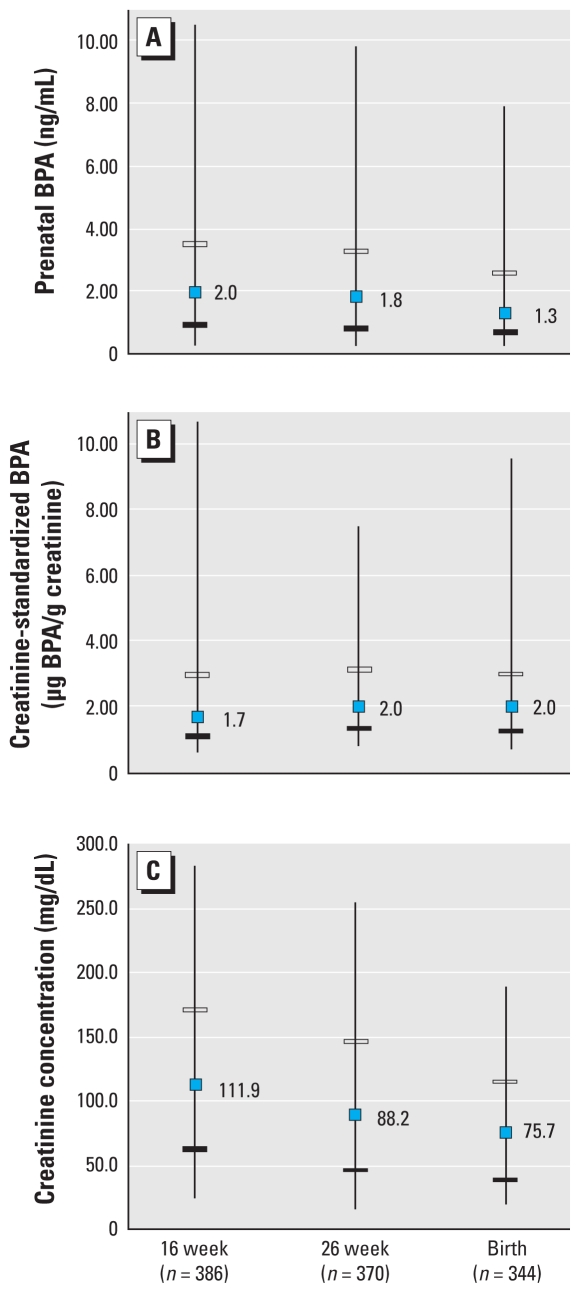
Distribution of prenatal BPA (*A*), creatinine-standardized BPA (*B*), and creatinine concentrations (*C*) during the latter two-thirds of pregnancy. Blue dots represent median values, black and white hatch marks the 25th and 75th percentiles, and whiskers the 5th and 95th percentiles.

On average (± SD), prenatal urine samples were collected at 16 ± 2.0, 26 ± 2.0, and 39 ± 1.8 weeks. Urine measurements at 39 weeks were taken around the time of delivery. More than 90% of women had detectable urinary BPA concentrations at 16 and 26 weeks of gestation, and 87.1% had detectable concentrations at birth. Unstandardized urinary BPA and creatinine concentrations decreased across pregnancy, but creatinine-standardized urinary BPA concentrations changed little over the latter two-thirds of gestation (Figure 1).

Log_10_-transformed urinary BPA concentrations were weakly correlated at 16 and 26 weeks (*r* = 0.28), 26 weeks and birth (*r* = 0.28), and 16 weeks and birth (*r* = 0.21). Correlations between creatinine-standardized urinary BPA concentrations were even lower at 16 and 26 weeks (*r* = 0.12), 26 weeks and birth (*r* = 0.12), and 16 weeks and birth (*r* = 0.06). There was no distinguishable pattern of correlations between either pair of urinary BPA measurements when we stratified by the time interval between collections.

The ICC for serial urinary BPA measurements indicated poor reproducibility in analyses using unstandardized (ICC = 0.25) and creatinine-standardized concentrations (ICC = 0.10). There was no substantial difference in the components of variance when we only used the 16-week and 26-week or the 26-week and birth concentrations (Table 1). Unstandardized and creatinine-standardized BPA concentrations varied by the time of day the urine sample was collected (Table 2). Creatinine-standardized BPA concentrations decreased across the morning hours, reaching a nadir of 1.7 μg/g between 1100 and 1259 hours, and increased in the afternoon, reaching a peak of 2.5 μg/g between 1500 and 1659 hours. Urinary creatinine concentrations were relatively stable in the early morning hours, decreased between 1500 and 1659 hours, and increased again between 1700 and 1900 hours.

Urinary BPA concentrations did not vary by most demographic factors, except for higher concentrations among women with lower education (≤ 12 years) compared with women with higher education (> 12 years; Table 3). In contrast, BPA concentrations varied by occupation. Prenatal urinary BPA concentrations were highest among women who reported being cashiers (GM = 2.8 μg/g; GSE = 1.1) and lowest among women who reported working in teaching (GM = 1.8 μg/g; GSE = 1.1) and industrial (GM = 1.2 μg/g; GSE = 1.2) occupations.

Frequency of canned vegetable consumption was positively associated with urinary BPA concentrations (Table 4). Strict vegetarians had lower urinary BPA concentrations compared with nonvegetarians, but estimates were based on a sample of only five women. Creatinine-standardized urinary BPA concentrations were similar among women who reported fasting for ≤ 12 hr but were lower among women who had been fasting > 12 hr (Table 2).

Urinary BPA concentrations were positively associated with serum cotinine concentrations (Table 5). Urinary concentrations of creatinine-standardized DEHP metabolite concentrations and high-molecular-weight phthalates were more positively associated with urinary BPA concentrations than concentrations of metabolites of low-molecular-weight phthalates (Table 5). Among individual phthalate metabolites, mono-2-ethylhexyl phthalate and mono-3-carboxypropyl phthalate had the strongest associations with creatinine-standardized urinary BPA concentrations [see Supplemental Material, Table 2 (doi:10.1289/ehp.1002366)].

Adjustment for socioeconomic factors did not appreciably change the association between dietary or environmental factors and urinary BPA concentrations (Tables 4 and 5). However, adjustment did attenuate the association between cashier work and urinary BPA concentrations [ratio = 1.15; 95% confidence interval (CI), 0.84–1.57]. This attenuation was due primarily to confounding by household income. Our results were not substantially different when we included log_10_-transformed urinary creatinine concentrations as a time-dependent covariate instead of creatinine-standardized BPA concentrations.

## Discussion

Serial urinary BPA concentrations were highly variable, had a low degree of reproducibility, and varied according to time of day of sample collection in the latter two-thirds of pregnancy. Occupational, dietary, and environmental factors were associated with urinary BPA concentrations. Working as a cashier, canned vegetables consumption, tobacco smoke exposure, and exposure to high-molecular-weight phthalates were positively associated with urinary BPA concentrations. Differences in prenatal urinary BPA concentrations among categories of some of these factors were of similar magnitude to differences in prenatal urinary BPA concentrations associated with externalizing behaviors in 2-year-old females in a prior study (Braun et al. 2009).

Our reported ICC (0.11) for repeated urinary BPA concentrations is lower than previous reports. Nepomnaschy et al. (2009) reported an ICC of 0.43 for three urinary BPA concentrations taken at 14-day intervals from 60 women of childbearing age. Teitelbaum et al. (2008) reported ICCs of 0.22–0.35 for urinary BPA concentrations among children 6–10 years of age over a 6-month period. Consistent with our findings, Adibi et al. (2008) reported a decreased ICC of urinary concentrations of phthalate metabolites in pregnant women when they adjusted for urine dilution using creatinine. Variations in the ICCs across studies could be related to differences in time between urine collections or increased creatinine excretion during pregnancy (Williams 2005).

Among demographic and perinatal factors, only maternal education was inversely associated with creatinine-standardized urinary BPA concentrations during pregnancy. A study using data from the National Health and Nutrition Examination Survey reported that income was inversely associated with urinary BPA concentrations (Calafat et al. 2008). However, two studies from China and the Netherlands documented higher urinary BPA concentrations among persons from higher social class (He et al. 2009; Ye et al. 2008). Women from lower social classes in the United States may consume more canned foods or live in neighborhoods where more canned fruits and vegetables are available than do women with higher socioeconomic status, but these relationships may be different in other countries (Morland and Filomena 2007). Furthermore, associations between urinary BPA concentrations and maternal education may be influenced by shared covariance with occupation or tobacco smoke exposure.

The frequency of consumption of canned vegetables, but not canned fruit, was positively associated with urinary BPA concentrations. We are not aware of any prior studies documenting this association, but this finding is not surprising because BPA can migrate from consumer goods into food and has been detected in canned foods (Cao et al. 2010; Kang and Kondo 2002; Thomson and Grounds 2005). A recent risk assessment suggests that canned vegetables contribute 10–40% of the daily BPA intake, whereas canned fruits contribute 3–6% (von Goetz et al. 2010). The relative contribution of canned vegetables to total BPA dose may vary according to the canning process, food variety, type of resin used, and, as shown here, frequency of consumption. Dietary patterns such as vegetarianism may influence BPA exposure, as suggested by the different concentrations among strict, partial, and nonvegetarians. However, we had a small number of women following a vegetarian diet, and the higher exposure among partial vegetarians is inconsistent with the lower concentration among strict vegetarians.

Compared with other occupations, cashiers had the highest urinary BPA concentrations. Most carbonless paper receipts used in convenience and grocery stores contain BPA, which could be dermally absorbed, orally ingested, or inhaled (vom Saal and Myers 2008). These results should be interpreted cautiously because estimates from cashiers were based on 17 women and were attenuated with adjustment for socioeconomic factors. Additional studies should validate our findings and, if they are validated, determine the primary route of exposure and if personal protective equipment (e.g., gloves) could prevent exposure.

Two common environmental exposures, phthalates and tobacco smoke, were positively associated with urinary BPA concentrations. Women with secondhand or active tobacco smoke exposure had urinary BPA concentrations about 20% higher than women with no tobacco smoke exposure. This finding is consistent with a prior study reporting higher urinary BPA concentrations among self-reported smokers (He et al. 2009). Inhaled and exhaled tobacco smoke may be a source of BPA because BPA comprises 25% of the weight of some cigarette filters (Jackson and Darnell 1985). Although socioeconomic factors may be partly responsible for the association between serum cotinine and urinary BPA concentrations, our estimates were not attenuated after adjustment for socioeconomic factors. Shared sources of BPA and high-molecular-weight phthalates, including DEHP, may be responsible for the positive correlation between urinary BPA and phthalate metabolite concentrations. These phthalates and BPA may be used in the same products (e.g., food packaging), whereas low-molecular-weight phthalates are used in cosmetics and beauty products (NRC 2008 ). Future studies should examine other potential sources of BPA and phthalate exposure.

These results have several implications for etiological studies of prenatal BPA exposure and health outcomes. Because urinary BPA concentrations varied according to the time of sample collection and fasting time, investigators should attempt to account for the inherent variability of urinary BPA concentrations. Future studies could use several approaches to reduce or adjust for this variability: *a*) standardize the timing of urine collection, *b*) collect multiple urine samples over the course of ≥ 1 days, or *c*) record and adjust for the time of day of sample collection. The low ICC for urinary BPA concentrations during pregnancy suggests that a single spot urine collection has the potential to misclassify exposure. Moreover, using mean BPA concentrations taken over the course of pregnancy may also result in exposure misclassification in studies attempting to identify time-sensitive windows of development to BPA exposure.

Studies examining the health impacts of prenatal BPA, phthalates, or tobacco smoke exposures may need to adjust for one another, because these pollutants frequency occur together and have been implicated in the etiology of childhood health outcomes (DiFranza et al. 2004; Engel et al. 2010; Wakschlag et al. 2002). In addition, future etiological studies should examine the joint effects of BPA, phthalates, and tobacco smoke exposure, because these common toxicants may occur together and act synergistically on certain health outcomes.

There are several limitations to this study. First, our results and others demonstrate that a single spot urine measurement has the potential to misclassify BPA exposure (Mahalingaiah et al. 2008). Second, many of our predictor variables were measured imperfectly, and we were missing some potentially important sources of exposure. We did not have women’s occupations classified by an industrial hygienist, which likely resulted in misclassification of this variable. Furthermore, the dietary variables used in this study were not originally designed to assess BPA exposure, but rather pesticide and mercury exposure. In addition, urinary BPA concentrations likely reflect exposure over the last day, whereas dietary questionnaire data reflected consumption over a longer time (weeks). Third, we did not collect information regarding other potential sources of BPA exposure including plastic or paper/cardboard use, packaged food consumption, medical devices, medications, dental treatment, or amount and type (tap, bottled, or well) of water consumed during pregnancy (Carwile et al. 2009; Gehring et al. 2004).

An additional limitation is the imperfect correction for urine dilution using urinary creatinine concentrations. Pregnancy-induced changes in creatinine metabolism and excretion may occur independently of BPA metabolism and excretion, so the degree of correction of urine dilution may change throughout pregnancy. Our results suggest that creatinine concentrations become progressively lower and more variable throughout pregnancy. Other measures of urine dilution, such as specific gravity, have been used and should be compared with creatinine patterns in pregnancy in future studies (Mahalingaiah et al. 2008).

A single spot urine sample may misclassify BPA exposure because of variability of urinary BPA concentrations over the course of pregnancy and day. Future studies should standardize or adjust for the timing of urine collection or measure BPA at multiple times to minimize biases due to within-day and within-woman variability of urinary BPA and creatinine concentrations. Our data suggest that there are numerous and potentially modifiable sources of environmental BPA exposure related to canned vegetable consumption, occupation, and other environmental exposures. Additional research is needed to confirm these findings and determine what other environmental sources contribute to human BPA exposure.

## Figures and Tables

**Table 1:** Proportion of variability in serial urinary BPA concentrations attributed to within-woman and between-woman variation.^a^

	Components of variance		
Outcome in mixed model	Between-woman (SE)	Within-woman (SE)	ICC^b^
Unstandardized BPA concentrations			

16 and 26 weeks	0.06 (0.01)	0.15 (0.01)	0.28
26 weeks and birth	0.05 (0.01)	0.15 (0.01)	0.26
16 weeks, 26 weeks, and birth	0.05 (0.01)	0.15 (0.01)	0.25

Creatinine-standardized BPA concentrations			

16 and 26 weeks	0.01 (0.01)	0.11 (0.01)	0.11
26 weeks and birth	0.01 (0.01)	0.10 (0.01)	0.12
16 weeks, 26 weeks, and birth	0.01 (0.00)	0.11 (0.01)	0.10

Creatinine concentrations			

16 and 26 weeks	0.06 (0.01)	0.06 (0.00)	0.49
26 weeks and birth	0.03 (0.01)	0.08 (0.01)	0.30
16 weeks, 26 weeks, and birth	0.04 (0.01)	0.08 (0.00)	0.35

a Includes only women who have all three urinary BPA measurements (*n* = 332).

b Computed by dividing the between-woman variation by the total variation (between-woman plus within-woman).

**Table 2:** GM mean urinary BPA and creatinine concentrations during pregnancy according to the time of urine collection and fasting time.

	*n*	*n*	BPA (μg/L)	Creatinine (mg/dL)	Creatinine-standardized BPA (μg/mg)
Variable	16 weeks	26 week	GM (GSE)	GM (GSE)	GM (GSE)
Time of day					

0700–0859	19	32	1.8 (1.2)	91.6 (1.1)	2.1 (1.1)
0900–1059	90	116	1.7 (1.1)	90.3 (1.1)	1.9 (1.1)
1100–1259	84	48	1.5 (1.1)	94.0 (1.1)	1.7 (1.1)
1300–1459	73	81	2.0 (1.1)	95.0 (1.1)	2.2 (1.1)
1500–1659	66	56	1.9 (1.1)	79.8 (1.1)	2.5 (1.1)
1700–1900	27	5	2.1 (1.2)	94.6 (1.1)	2.3 (1.2)

Fasting time					

0 to < 2 hr	51	47	1.7 (1.7)	85.7 (1.1)	2.1 (1.1)
2 to < 4 hr	83	90	1.6 (1.6)	83.8 (1.1)	2.1 (1.1)
4 to < 6 hr	131	107	1.7 (1.7)	86.9 (1.1)	2.1 (1.1)
6 to < 12 hr	45	30	2.9 (2.9)	130.6 (1.1)	2.1 (1.1)
12 to 24 hr	22	26	1.9 (1.9)	104.7 (1.1)	1.8 (1.1)

Log_10_-transformed urinary BPA, creatinine, or creatinine-standardized BPA concentrations at 16 and 26 weeks of gestation are the outcome in a linear mixed model.

**Table 3:** GM urinary BPA concentrations (μg BPA/g creatinine) according to demographic, perinatal, and maternal factors.^a^

Variable	*n* (%)^b^	GM (GSE)	Ratio (95% CI)
Maternal race			
Non-Hispanic white	237 (62)	2.1 (1.0)	Reference
Non-Hispanic black	120 (31)	2.1 (1.0)	1.01 (0.90–1.14)
Other	26 (7)	1.9 (1.1)	0.94 (0.75–1.16)
Maternal education (years)			
> 12	288 (75)	2.0 (1.0)	Reference
12	54 (14)	2.3 (1.1)	1.14 (0.98–1.33)
< 12	41 (11)	2.4 (1.1)	1.19 (1.00–1.41)
Marital status			
Married	248 (65)	2.0 (1.0)	Reference
Unmarried	134 (35)	2.2 (1.0)	1.08 (0.96–1.20)
Maternal age (years)			
25–34	230 (59)	2.0 (1.0)	Reference
< 25	96 (25)	2.1 (1.1)	1.05 (0.92–1.19)
> 35	62 (16)	2.0 (1.1)	0.97 (0.84–1.12)
Income (per year)			
> $80,000	103 (28)	2.0 (1.1)	Reference
$40,000 to < $80,000	120 (32)	2.0 (1.0)	1.04 (0.91–1.19)
$20,000 to < $40,000	65 (17)	2.0 (1.1)	1.02 (0.86–1.20)
< $20,000	87 (23)	2.2 (1.1)	1.13 (0.97–1.31)
Depression Score at 20 weeks			
Minimal (< 13)	290 (78)	2.1 (1.0)	Reference
Moderate (13–19)	54 (14)	2.1 (1.1)	1.01 (0.86–1.18)
Severe (> 19)	30 (8)	1.9 (1.1)	0.90 (0.74–1.09)
Parity			
0	171 (44)	2.1 (1.0)	Reference
1	123 (32)	2.0 (1.0)	0.97 (0.86–1.09)
> 1	92 (24)	2.1 (1.1)	0.98 (0.86–1.12)
Maternal IQ (per 10 points)	318	—	0.97 (0.93–1.01)
Child sex			
Female	208 (54)	2.1 (1.0)	Reference
Male	180 (46)	2.0 (1.0)	0.96 (0.86–1.06)

a Ratios are the exponentiated beta coefficients from a linear mixed model with 16-week, 26-week, and birth creatinine-standardized BPA measurements as the outcome. Ratios represent the multiplicative difference in creatinine-standardized urinary BPA concentrations from the reference category. Each predictor is run in a separate model.

b At 16-week visit.

**Table 4:** GM urinary BPA concentrations (μg BPA/g creatinine) according to dietary and occupational factors.^a^

Variable	*n* (%)^b^	GM (GSE)	Unadjusted ratio (95% CI)	Adjusted ratio (95% CI)^c^
Frequency of fish consumption				

Not at all	57 (15)	2.1 (1.1)	Reference	Reference
< Once/month	113 (30)	2.1 (1.0)	1.01 (0.86–1.18)	1.03 (0.88–1.20)
1–3 times/month	131 (34)	2.0 (1.0)	0.94 (0.80–1.10)	0.97 (0.83–1.13)
Weekly or more	77 (19)	2.1 (1.1)	1.00 (0.84–1.20)	1.04 (0.87–1.24)

Frequency of canned fruit consumption				

Not at all	45 (12)	2.0 (1.1)	Reference	Reference
< Once/month	55 (14)	2.0 (1.1)	1.04 (0.86–1.26)	1.04 (0.85–1.26)
1–3 times/month	91 (24)	2.0 (1.1)	1.03 (0.86–1.22)	1.01 (0.85–1.21)
1–3 times/week	96 (25)	2.1 (1.1)	1.05 (0.89–1.25)	1.05 (0.88–1.25)
4–6 times/week	50 (13)	2.2 (1.1)	1.13 (0.92–1.39)	1.13 (0.92–1.39)
≥ Once/day	45 (12)	2.2 (1.1)	1.09 (0.89–1.34)	1.08 (0.88–1.33)

Frequency of canned vegetable consumption				

Not at all	17 (4)	1.6 (1.1)	Reference	Reference
< Once/month	30 (8)	2.0 (1.1)	1.24 (0.90–1.69)	1.42 (1.03–1.97)
1–3 times/month	58 (15)	2.1 (1.1)	1.29 (0.97–1.71)	1.44 (1.07–1.93)
1–3 times/week	133 (35)	2.0 (1.0)	1.22 (0.93–1.59)	1.38 (1.04–1.83)
4–6 times/week	88 (23)	2.2 (1.1)	1.38 (1.05–1.82)	1.56 (1.17–2.09)
≥ Once/day	56 (15)	2.3 (1.1)	1.39 (1.04–1.86)	1.52 (1.13–2.06)

Frequency of fresh fruit and vegetable consumption				

> Once/day	77 (20)	2.0 (1.0)	Reference	Reference
About once a day	73 (19)	1.7 (1.2)	0.84 (0.57–1.22)	1.02 (0.88–1.19)
4–6 times/week	104 (27)	2.2 (1.1)	1.09 (0.94–1.25)	1.07 (0.93–1.23)
1–3 times/week	83 (22)	2.0 (1.1)	0.97 (0.84–1.13)	0.96 (0.82–1.12)
1–3 times/month	39 (10)	2.2 (1.1)	1.09 (0.89–1.35)	1.03 (0.83–1.29)
< Once/month or not at all	6 (2)	2.4 (1.2)	1.22 (0.84–1.76)	1.19 (0.80–1.77)

Vegetarian				

No	363 (95)	2.1 (1.0)	Reference	Reference
Partial	14 (4)	2.4 (1.1)	1.16 (0.88–1.52)	1.17 (0.89–1.55)
Strict	5 (1)	1.3 (1.3)	0.64 (0.41–1.00)	0.64 (0.40–1.00)

Organic fruit and vegetable consumption				

None	225 (59)	2.1 (1.0)	Reference	Reference
Less than half	122 (32)	2.1 (1.1)	1.00 (0.89–1.12)	1.03 (0.91–1.16)
More than Half	32 (8)	2.0 (1.1)	0.99 (0.81–1.21)	0.98 (0.79–1.21)

Occupation				

Unemployed	84 (22)	1.9 (1.1)	Reference	Reference
Cashier	17 (4)	2.8 (1.1)	1.32 (1.00–1.75)	1.15 (0.84–1.57)
Health care worker	57 (15)	2.1 (1.1)	1.00 (0.84–1.19)	1.04 (0.87–1.25)
Food service	16 (4)	2.1 (1.2)	1.01 (0.74–1.39)	1.02 (0.74–1.40)
Industrial worker	14 (4)	1.2 (1.2)	0.59 (0.40–0.87)	0.61 (0.41–0.90)
Teacher	37 (10)	1.8 (1.1)	0.84 (0.69–1.03)	0.89 (0.71–1.12)
Office worker	118 (31)	2.1 (1.0)	1.00 (0.87–1.15)	1.06 (0.91–1.24)
Sales or service worker	36 (9)	2.1 (1.1)	1.00 (0.82–1.21)	1.04 (0.85–1.28)
Other	2 (1)	1.2 (1.4)	0.59 (0.31–1.14)	0.60 (0.31–1.18)

a Ratios are the exponentiated beta coefficients from a linear mixed model with 16-week, 26-week, and birth creatinine-standardized BPA measurements as the outcome. Ratios represent the multiplicative difference in creatinine-standardized urinary BPA concentrations from the reference category. Each predictor is run in a separate model.

b At 16-week visit.

c Adjusted for maternal age, race, education, household income, and marital status.

**Table 5:** GM urinary BPA concentrations (μg BPA/g creatinine) by biomarkers of tobacco smoke and phthalates exposure.^a^

Variable	*n* (%)^b^	GM (GSE)	Unadjusted ratio (95% CI)	Adjusted ratio (95% CI)^c^
Categorical serum cotinine concentrations				

Unexposed (< 0.015 ng/mL)	112 (30)	1.9 (1.0)	Reference	Reference
Secondhand smoker (0.015–3 ng/mL)	221 (59)	2.2 (1.0)	1.17 (1.05–1.30)	1.19 (1.05–1.35)
Active smoker (> 3 ng/mL)	43 (11)	2.3 (1.1)	1.23 (1.03–1.47)	1.27 (1.01–1.58)

Phthalates^d^				

Low-molecular-weight phthalates	387	—	1.07 (0.97–1.18)	1.06 (0.96–1.18)
High-molecular-weight phthalates	387	—	1.28 (1.16–1.40)	1.26 (1.15–1.39)
DEHP metabolites	387	—	1.24 (1.14–1.36)	1.24 (1.13–1.35)

a Ratios are the exponentiated beta coefficients from a linear mixed model with 16-week, 26-week, and birth creatinine-standardized BPA measurements as the outcome. Ratios represent the multiplicative difference in creatinine-standardized urinary BPA concentrations from the reference category. Each predictor is run in a separate model.

b At 16-week visit.

c Adjusted for maternal age, race, education, household income, and marital status.

d Low-molecular-weight phthalates include MBP (monobutyl phthalate), MEP (monoethyl phthalate), and MIBP (monoisobutyl phthalate). High-molecular-weight phthalates include MBzP (monobenzyl phthalate), MEHP (mono-2-ethylhexyl phthalate), MECPP [mono-(2-ethyl-5-carboxypentyl) phthalate], MEHHP [mono-(2-ethyl-5-hydroxylhexyl) phthalate], MEOHP [mono-(2-ethyl-5-oxohexyl) phthalate], and MCPP (mono-3-carboxypropyl phthalate). DEHP metabolites include MECPP, MEHHP, MEOHP, and MEHP. All phthalates concentrations are creatinine standardized.
